# Environmental toxicant exposure and sleep: Findings from a U.S. population study

**DOI:** 10.1192/j.eurpsy.2026.11990

**Published:** 2026-07-31

**Authors:** S. Leng, Y. Jin, X. Tang

**Affiliations:** West China Hospital, Sichuan University, Chengdu, China

## Abstract

**Introduction:**

Sleep plays a vital role in promoting health. Elucidating the associations between common environmental toxicants and sleep is essential to guide targeted mechanistic studies and inform preventive strategies.

**Objectives:**

This study aims to systematically evaluate the relationships between a broad spectrum of environmental toxicants and both sleep disturbances and sleep duration.

**Methods:**

We analyzed data from participants in the 2013–2014 and 2015–2016 waves of the National Health and Nutrition Examination Survey (NHANES) with available measurements of blood or urinary environmental toxicants, sleep disturbances, and sleep duration. A total of 82 toxicants spanning 13 categories were examined, including aromatic amines, arsenic species, bisphenols, heterocyclic aromatic amines, iodine, metals, polycyclic aromatic hydrocarbons (PAHs), volatile organic compound (VOC) metabolites, per- and polyfluoroalkyl substances (PFAS), phthalates, parabens, perchlorate, nitrate, thiocyanate, pyrethroids, herbicides, and organophosphate (OP) metabolites. An exposome-wide association study and the deletion-substitution-addition (DSA) algorithm were used to assess associations with self-reported **sleep disturbance** and duration adjusted for important covariates. Subgroup analyses stratified by sex and age were performed.

**Results:**

Among 3,882 adults included (mean age 45.8 ± 19.0 years; 1,907 [49.1%] female), five categories of environmental toxicants (PAHs, VOCs, metals, OP metabolites, and thiocyanate) were significantly associated with sleep disturbance prevalence. Subgroup analyses revealed that females and younger individuals were more vulnerable to these toxicants than their male and older counterparts. Comparably, several environmental toxicants were associated with abnormal sleep duration, with certain pyrethoids, VOCs, parabens, and metals linked to short sleep duration (< 6 hours) and certain pyrethoids, bisphenols, and PFAS to long sleep duration (> 10 hours).

**Image 1:**
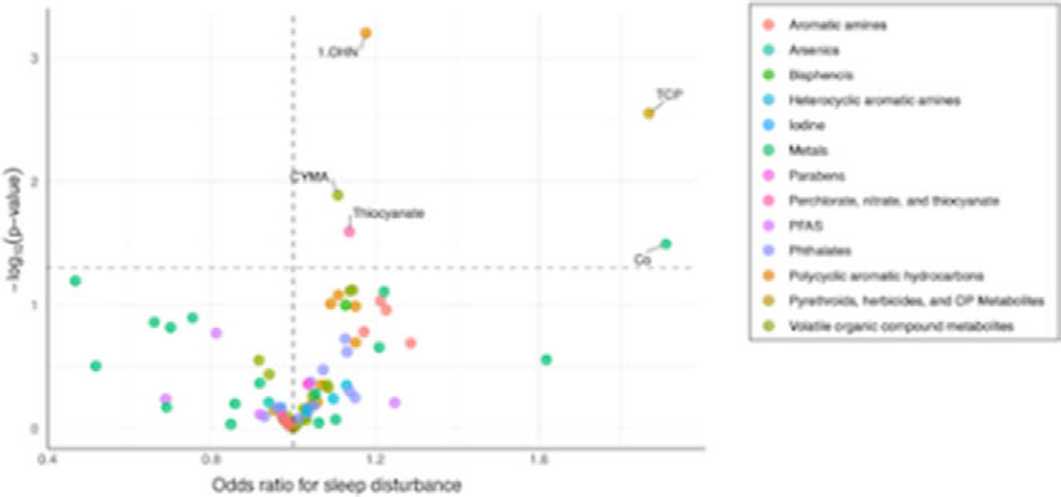
Image 1: Long description.

**Image 2:**
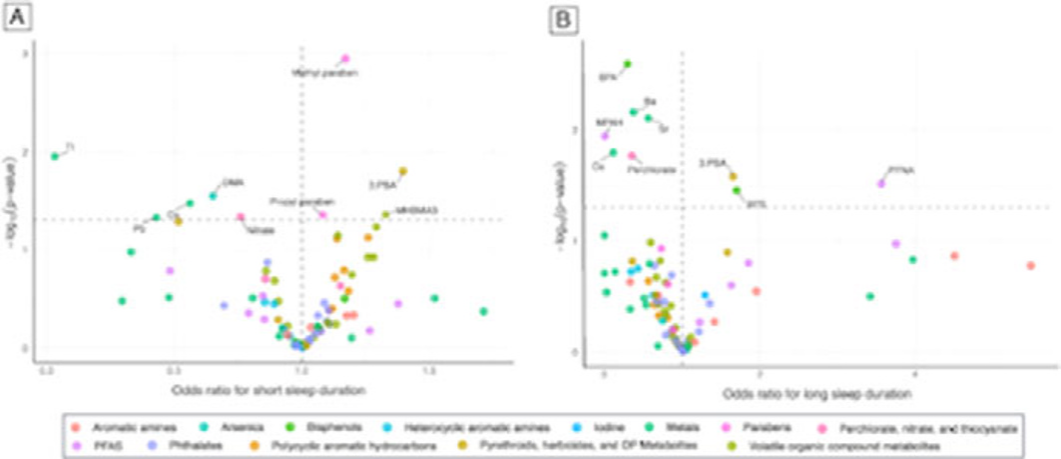
Image 2: Long description.

**Image 3:**
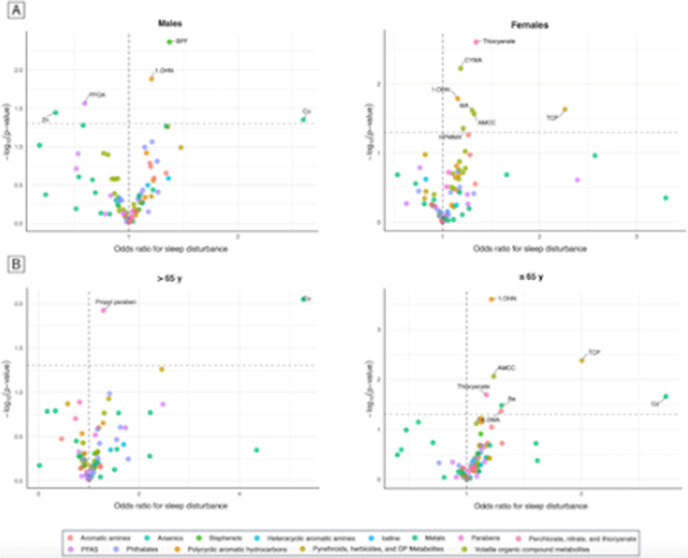
Image 3: Long description.

**Conclusions:**

Our findings demonstrate that multiple categories of environmental toxicants are associated with both sleep disturbances and sleep duration. This study offers important insights for prioritizing environmental targets in mechanistic research on sleep regulation and supports efforts to mitigate harmful exposures.

**Disclosure of Interest:**

None Declared

## Long descriptions

### Image 1: Long description

The X-axis represents the Odds ratio for sleep disturbance, ranging from 0.4 to 2.0, with a vertical dashed line at 1.0. The Y-axis represents minus log sub 10 p-value, ranging from 0 to 3, with a horizontal dashed line at approximately 1.3.

Data points are color-coded by chemical category, including Aromatic amines, Arsenics, Bisphenols, Heterocyclic aromatic amines, Iodine, Metals, Parabens, Perchlorate, nitrate, and thiocyanate, P F A S, Phthalates, Polycyclic aromatic hydrocarbons, Pyrethroids, herbicides, and O P Metabolites, and Volatile organic compound metabolites.

Significant outliers in the upper quadrants include:

* 1-O H N (Polycyclic aromatic hydrocarbon) at the top center with the highest significance (Y > 3).

* T C P (Pyrethroids, herbicides, and O P Metabolites) in the upper right quadrant (X approx 1.9, Y approx 2.5).

* C Y M A (Volatile organic compound metabolite) just above the horizontal threshold near the center.

* Thiocyanate (Perchlorate, nitrate, and thiocyanate) just below C Y M A.

* C o (Metals) on the far right, sitting on the horizontal significance threshold.

The majority of data points are clustered below the horizontal significance threshold (Y < 1.3) and between odds ratios of 0.8 and 1.2.

Navigate back to Image 1:.

### Image 2: Long description

A two-panel figure labeled A and B. Both panels are volcano plots where the x-axis represents the Odds ratio and the y-axis represents minus log sub 10 p-value. A horizontal dashed line indicates statistical significance at p equals 0.05.

Panel A is titled Odds ratio for short sleep-duration. The data points form a V-shape centered at an Odds ratio of 1.0. Labeled points above the significance threshold include Methyl paraben at the highest significance, followed by T l, 3-P S A, D M A, Propyl paraben, Nitrate, and M H B M A S.

Panel B is titled Odds ratio for long sleep-duration. The distribution is skewed with more significant points on the left side (Odds ratio less than 1). Labeled points include B P A, B a, M P B H, S r, C o, Perchlorate, 3-P S A, and A E T L on the left, and P F N A on the right.

A shared legend at the bottom categorizes the points by color:

- Aromatic amines (coral)

- Arsenics (teal)

- Bisphenols (green)

- Heterocyclic aromatic amines (light blue)

- Iodine (blue)

- Metals (seafoam green)

- Parabens (light pink)

- Perchlorate, nitrate, and thiocyanate (dark pink)

- P F A S (light purple)

- Phthalates (periwinkle)

- Polycyclic aromatic hydrocarbons (orange)

- Pyrethroids, herbicides, and D P Metabolites (gold)

- Volatile organic compound metabolites (olive green).

Navigate back to Image 2:.

### Image 3: Long description

Four scatter plots arranged in a two-by-two grid labeled A and B. All plots share the same axes. The x-axis represents Odds ratio for sleep disturbance, and the y-axis represents minus log sub 10 p-value. A vertical dashed line is at x equals 1, and a horizontal dashed line is at y equals 1.3.

Top-left panel (Males): Data points are clustered near the origin. Significant outliers include B P F and 1-O H N in the upper-middle region, P F O A in the upper-left, and C d in the upper-right.

Top-right panel (Females): Shows a higher density of points above the horizontal threshold compared to males. Notable outliers include Thiocyanate at the top, followed by C Y M A, 1-O H N, M A, and A M C C. T C P is an outlier on the far right.

Bottom-left panel (greater than 65 y): Most points are below the significance threshold. Propyl paraben is a high outlier on the left, and C d is a high outlier on the far right.

Bottom-right panel (less than or equal to 65 y): Shows several significant outliers including 1-O H N at the top, followed by A M C C, T C P, Thiocyanate, S e, and C d.

A legend at the bottom categorizes points by color: Aromatic amines (coral), Arsenics (teal), Bisphenols (bright green), Heterocyclic aromatic amines (light blue), Iodine (blue), Metals (green), Parabens (pink), Perchlorate, nitrate, and thiocyanate (magenta), P F A S (purple), Phthalates (lavender), Polycyclic aromatic hydrocarbons (orange), Pyrethroids, herbicides, and O P Metabolites (gold), and Volatile organic compound metabolites (olive).

Navigate back to Image 3:.

